# Location-specific ASPECTS does not improve Outcome Prediction in Large Vessel Occlusion compared to Cumulative ASPECTS

**DOI:** 10.1007/s00062-022-01258-8

**Published:** 2023-01-26

**Authors:** Ulf Neuberger, Dominik F. Vollherbst, Christian Ulfert, Silvia Schönenberger, Christian Herweh, Simon Nagel, Peter A. Ringleb, Markus A. Möhlenbruch, Martin Bendszus, Philipp Vollmuth

**Affiliations:** 1 Dept. of Neuroradiology, Heidelberg University Hospital, Im Neuenheimer Feld 400, 69120 Heidelberg, Germany; 2 Dept. of Neurology, Heidelberg University Hospital, Heidelberg, Germany; 3 Department of Neurology, Städtisches Klinikum Ludwigshafen, Ludwigshafen, Germany

**Keywords:** Stroke, Thrombectomy, ASPECTS, Machine-learning, Computed tomography

## Abstract

**Purpose:**

Individual regions of the Alberta Stroke Programme Early CT Score (ASPECTS) may contribute differently to the clinical symptoms in large vessel occlusion (LVO). Here, we investigated whether the predictive performance on clinical outcome can be increased by considering specific ASPECTS subregions.

**Methods:**

A consecutive series of patients with LVO affecting the middle cerebral artery territory and subsequent endovascular treatment (EVT) between January 2015 and July 2020 was analyzed, including affected ASPECTS regions. A multivariate logistic regression was performed to assess the individual impact of ASPECTS regions on good clinical outcome (defined as modified Rankin scale after 90 days of 0–2). Machine-learning-driven logistic regression models were trained (training = 70%, testing = 30%) to predict good clinical outcome using i) cumulative ASPECTS and ii) location-specific ASPECTS, and their performance compared using deLong’s test. Furthermore, additional analyses using binarized as well as linear clinical outcomes using regression and machine-learning techniques were applied to thoroughly assess the potential predictive properties of individual ASPECTS regions and their combinations.

**Results:**

Of 1109 patients (77.3 years ± 11.6, 43.8% male), 419 achieved a good clinical outcome and a median NIHSS after 24 h of 12 (interquartile range, IQR 4–21). Individual ASPECTS regions showed different impact on good clinical outcome in the multivariate logistic regression, with strongest effects for insula (odds ratio, OR 0.56, 95% confidence interval, CI 0.42–0.75) and M5 (OR 0.53, 95% CI 0.29–0.97) regions. Accuracy (ACC) in predicting good clinical outcome of the test set did not differ between when considering i) cumulative ASPECTS and ii) location-specific ASPECTS (ACC = 0.619, 95% CI 0.58–0.64 vs. ACC = 0.629, 95% CI 0.60–0.65; *p* = 0.933).

**Conclusion:**

Cumulative ASPECTS assessment in LVO remains a stable and reliable predictor for clinical outcome and is not inferior to a weighted (location-specific) ASPECTS assessment.

**Supplementary Information:**

The online version of this article (10.1007/s00062-022-01258-8) contains supplementary material, which is available to authorized users.

## Introduction

Brain imaging is a key criterion to select patients with acute ischemic stroke (AIS) due to large vessel occlusion (LVO) for endovascular therapy (EVT). In the presence of LVO affecting the middle cerebral artery (MCA) territory, early signs of ischemia are usually quantified by applying the Alberta Stroke Programme Early CT Score (ASPECTS), subdividing the MCA territory into 10 different predefined regions [1]. ASPECTS has widely been used as selection criterion for studies in ischemic stroke [2], however, in clinical practice there is no uniformly defined cut-off for treatment decisions in individual patients. Even though lower ASPECTS are commonly associated with a poorer chance for a good outcome, patients with supposedly low baseline ASPECTS (< 6) can still achieve good functional outcome after complete recanalization [3]. Likewise, it was also observed that patients with a relatively high ASPECTS might achieve a poor long-term clinical outcome, even despite successful recanalization. This circumstance inevitably results in patients being denied treatment due to an ASPECTS that is supposedly too low, even though they could still benefit from EVT.

A potential explanation for this seemingly apparent contradiction may be that the involvement of specific anatomical areas in ischemic stroke is associated with poorer functional outcome, e.g., the primary motor cortex or areas linked to language processing [4]; however, all 10 ASPECTS areas are considered as equally important. Additional factors that limit the information value of the ASPECTS are the disregard of the different sizes of the ASPECTS regions as well as the laterality of stroke, which also has been shown to play a crucial role in outcome prediction [5]. To sharpen the prognostic value of ASPECTS, previous studies suggested a differing impact for some ASPECTS subregions on predicting EVT outcomes [6, 7]; however, the clinical value of a weighted, location-specific ASPECTS as compared to the established cumulative ASPECTS has not yet been investigated.

In the present study, we compared the performance of the cumulative vs. location-specific ASPECTS with differential weighting of the affected regions for predicting EVT outcome in AIS.

## Methods

### Patients and Study Design

We reviewed consecutive patients with AIS undergoing EVT between January 2015 and July 2020 from a prospectively compiled database of a tertiary care university hospital to identify all patients with LVO affecting the MCA territory. Clinically and procedure-related parameters of patients are presented in Table 1.

**Table 1:** Baseline characteristics as well as procedural and clinical results of all included patients (*n* = 1109)

Parameter	All patients (*n* = 1109)
*Baseline characteristics*	
Age, years	76.0 ± 12
Sex, male	484 (44)
Hypertension	858 (77)
Diabetes mellitus	245 (22)
Atrial fibrillation	527 (48)
Dyslipidemia	346 (31)
Coronary heart disease	285 (26)
Prestroke RS	1 (0–2)
*Baseline measurements*	
NIHSS score	16 (11–21)
Systolic blood pressure, mm Hg	145.9 ± 30
Diastolic blood pressure, mm Hg	79.3 ± 21
Serum glucose, mg/dL	128.5 ± 44
HbA1c (%)	6.0 ± 1.0
*Procedural and clinical results*	
ASPECTS in baseline imaging	9 (8–10)
Prevalence of cortical lesions (M1–M6 regions)	104 (28)
Stroke of right hemisphere	531 (48)
Occlusion of distal ICA	82 (7)
Occlusion of carotid T	213 (19)
Occlusion of proximal MCA	576 (52)
Occlusion of distal M1 or proximal M2	288 (26)
i.v. rtPA	567 (51)
Onset to groin puncture, min	257 (176–429)
Groin puncture to recanalization, min	26 (18–37)
Procedure length, min	54 (34–94)
Fluoroscopy time, min	20 (13–41)
Stent-retriever maneuvers	2.1 ± 1.9
Aspiration maneuvers	0.5 ± 0.9
Direct aspiration first-pass technique	431 (39)
Severe stenosis of ICA	225 (20)
Acute stenting of ICA	135 (12)
mTICI 3	373 (34)
mTICI 2c	170 (15)
Complete recanalization (mTICI 2c–3)	543 (49)
Failed recanalization (mTICI 0–1)	68 (6)
ASPECTS in follow-up imaging	7 (5–8)
Symptomatic intracranial hemorrhage in follow-up imaging	35 (3)
Rate of progression of ischemic lesions (follow-up ASPECTS < baseline ASPECTS)	762 (69)
NIHSS after 24 h	12 (4–21)
Shift of NIHSS after 24 h	3 (2–9)
NIHSS at discharge	3 (1–10)
mRS at discharge	4 (2–5)
mRS after 90 days	4 (2–5)
Mortality after 90 days	245 (22)

Values are given as mean ± SD or *n* (%) or median (interquartile range)

*mRS* modified Rankin scale, *NIHSS* National Institutes of Health Stroke Scale, *ASPECTS* Alberta Stroke Programme Early CT Score, *MCA* middle cerebral artery, *ICA* internal carotid artery, *i.v. rtPA* concomitant treatment with intravenous recombinant tissue-type plasminogen activator, *mTICI* modified thrombolysis in cerebral infarction scale

Because of its retrospective character, additional written informed consent was waived by the local ethics committee.

### Imaging, Endovascular Treatment and Clinical Assessment

All patients received non-contrast cranial computed tomography (CT) supplemented by CT angiography on admission and subsequent EVT. Patients with a suspected time window of more than 6 h since the onset of the stroke additionally received CT perfusion. ASPECTS, including its subregions, were evaluated visually by a board-certified radiologist with 8 years of experience (UN) in consensus with automated analysis by e‑ASPECTS (Brainomix, Oxford, UK) on 1 mm slices [8, 9]. Baseline imaging was acquired at admission and the decision for endovascular treatment as well as the administration and dosing of rtPA was individually made for each patient based on a consensus between the treating neurologist and neurointerventionalist, following national and international guidelines. Success of recanalization was evaluated by applying the modified thrombolysis in cerebral infarction (mTICI, with mTICI 2b or 3 representing complete recanalization) scale by the treating neurointerventionalist [10]. Routine follow-up cranial CT or magnetic resonance imaging (MRI) was performed within 18–36 h or earlier in cases of clinical deterioration and visually assessed by follow-up ASPECTS. Clinical symptoms were assessed according to the National Institutes of Health Stroke Scale (NIHSS) score on admission and 24 h after EVT, as well as modified Rankin Scale (mRS) at 90 days after onset by certified neurologists blinded to the intervention. A NIHSS score of 42 was assigned to patients who died during the initial hospitalization.

### Statistics

Statistical analyses were performed using R version 4.0.3 (R Foundation for Statistical Computing, Vienna, Austria).

Descriptive statistics of clinical and imaging data as mean (± standard deviation), *n* (%) or median (interquartile range, IQR) were calculated, accordingly (see Table 1).

A multivariate logistic regression model was built using each ASPECTS region as binary value, as well as onset-to-imaging time as continuous value, as independent parameters, and good clinical outcome (defined as mRS of 0–2 after 90 days) as dependent variable. This experiment served as a preliminary investigation of whether different effects of the individual regions on clinical outcome, as observed in other publications, were derivable in our patient cohort.

Multivariate logistic regression models were built using each ASPECTS region as binary value for the outcomes of i) mRS 0–1 after 90 days, ii) mRS 0–2 after 90 days, iii) mRS 0–3 after 90 days, iv) mRS 4–6 after 90 days and v) mRS 6 after 90 days. For all models, the logistic regression coefficients of each ASPECTS region were extracted and then applied to each region as a weighting factor to derive an adjusted ASPECTS (aASPECTS). To ensure comparability between the original ASPECTS (oASPECTS) and the aASPECTS, the scale of the aASPECTS was normalized to 10 points. Univariate logistic regression models for both i) oASPECTS and ii) aASPECTS were then performed for the respective outcome, in which the weighting factors were initially obtained, and effects on the outcome measured by odds ratios and their corresponding 95% confidence intervals of the models were compared.

Details about the comparison of cumulative and location-specific ASPECTS using machine-learning driven predictive models on clinical outcome can be found in the online-only supplemental material.

### Supplemental Analyses

Additional analyses were performed to investigate the predictive implications of ASPECTS subregions and their combinations. For this purpose, additional binary as well as linear outcomes on the mRS and NIHSS spectrum were analyzed using multivariate logistic and linear regressions as well as machine-learning approaches, including the comparison of different machine-learning algorithms. Furthermore, subgroup analyses were performed to assess the effects of laterality as well as complete recanalization. Details of these additional analyses are provided in the online-only supplemental material.

## Results

**Fig. 1:**
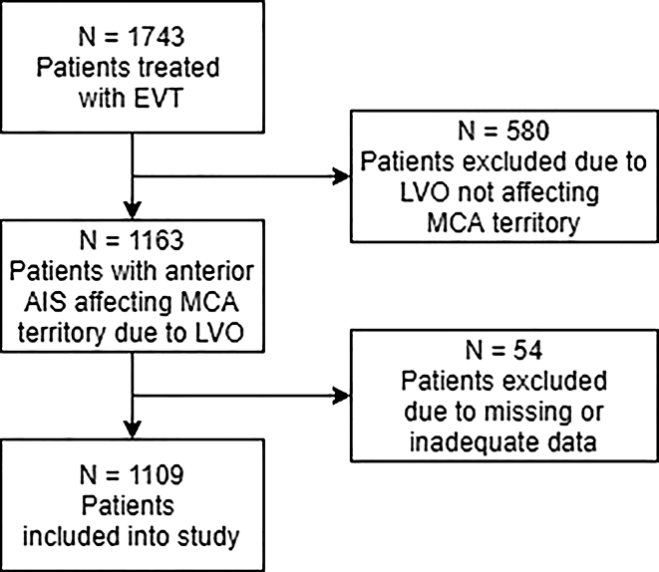
Flowchart of patient selection. *EVT* endovascular treatment, *MCA* middle cerebral artery, *LVO* large vessel occlusion, *AIS* acute ischemic stroke

A total of 1109 patients met the inclusion criteria (see Fig. 1 for a flowchart of patient selection). The median NIHSS was 16 (IQR 11–21) on admission and 12 (IQR 4–21) after 24 h. In 576 cases (51.9%), the proximal M1 segment was occluded, while in 288 cases (26.0%) either distal M1 or proximal M2 occlusions were present. Complete recanalization was achieved in 543 patients (49.0%), 90 days after treatment, the median mRS was 3 (IQR 2–5), with 415 patients (37.4%) achieving a good clinical outcome, 545 patients (49.1%) had an unfavorable outcome (mRS 4–6), with a total of 245 fatal cases (22.1%). An overview of clinically and procedure-related parameters of patients is presented in Table 1.

### Predicting Clinical Outcome Using Logistic Regression Models

Multivariate regression with all ASPECTS subregions for good clinical outcome showed strongest effects for insula (OR 0.56, 95% CI 0.42–0.75) and M5 (OR 0.53, 95% CI 0.29–0.97). An overview of the adjusted effects of all regions on good as well as poor clinical outcome can be found in Table 2. Here, the insular cortex as well as the M5 region showed significant associations to good clinical outcome.

**Table 2:** Location-specific subregions and cumulative ASPECTS at baseline for patients with good outcome (mRS of 0–2 after 90 days) and poor outcome (mRS of 0–3 after 90 days)

ASPECTS regions	Good outcome (*n* = 415)	Poor outcome (*n* = 694)	OR (95% CI)
Caudate, *n* (%)	87 (20.1)	195 (47.0)	0.86 (0.62–1.20)
Putamen, *n* (%)	137 (33.0)	298 (71.8)	0.78 (0.58–1.01)
Internal capsule, *n* (%)	6 (1.4)	35 (5.0)	0.79 (0.15–4.25)
Insular cortex, *n* (%)	128 (31.0)	357 (86.0)	0.56 (0.42–0.75)
M1, *n* (%)	27 (6.5)	100 (24.1)	0.86 (0.51–1.46)
M2, *n* (%)	45 (10.8)	163 (39.3)	0.86 (0.55–1.35)
M3, *n* (%)	11 (2.7)	44 (10.6)	0.76 (0.36–1.61)
M4, *n* (%)	10 (2.4)	56 (13.5)	0.57 (0.26–1.24)
M5, *n* (%)	19 (4.6)	102 (24.6)	0.53 (0.29–0.97)
M6, *n* (%)	13 (3.1)	47 (11.3)	0.97 (0.46–2.02)
Cumulative ASPECTS, median (IQR)	9 (8–10)	8 (7–10)	n. a.

*ASPECTS* Alberta Stroke Programme Early CT Score, *mRS* modified Rankin Scale, *IQR* interquartile range, *OR* odds ratios, *CI* confidence interval

Results of the comparison of location-specific ASPECTS, applying regional weighting factors derived by logistic regression, and the cumulative ASPECTS on good clinical outcome are summarized in supplemental Table 3.

**Table 3:** Development of an adjusted ASPECTS by means of logistic regression and comparison to the original ASPECTS on clinical outcome for all patients (*n* = 1109). Results are given in OR and 95% CI. No significant differences on clinical outcomes between the respective models, indicated by the overlapping confidence intervals, were observed

Outcome definition (binarized)	OR (95% CI) of original ASPECTS	OR (95% CI) of weighted ASPECTS
mRS 0–1 after 90 days	1.44 (1.30–1.60)	1.34 (1.23–1.46)
mRS 0–2 after 90 days	1.38 (1.27–1.50)	1.31 (1.22–1.40)
mRS 0–3 after 90 days	1.25 (1.16–1.34)	1.18 (1.12–1.24)
mRS 4–6 after 90 days	0.74 (0.68–0.81)	0.76 (0.70–0.82)
mRS 6 after 90 days	0.84 (0.78–0.91)	0.89 (0.85–0.93)

*ASPECTS* Alberta Stroke Programme Early CT Score, *mRS* modified Rankin Scale, *OR* odds ratios, *CI* confidence interval

### Predicting Clinical Outcome Using Machine-learning Models

**Fig. 2:**
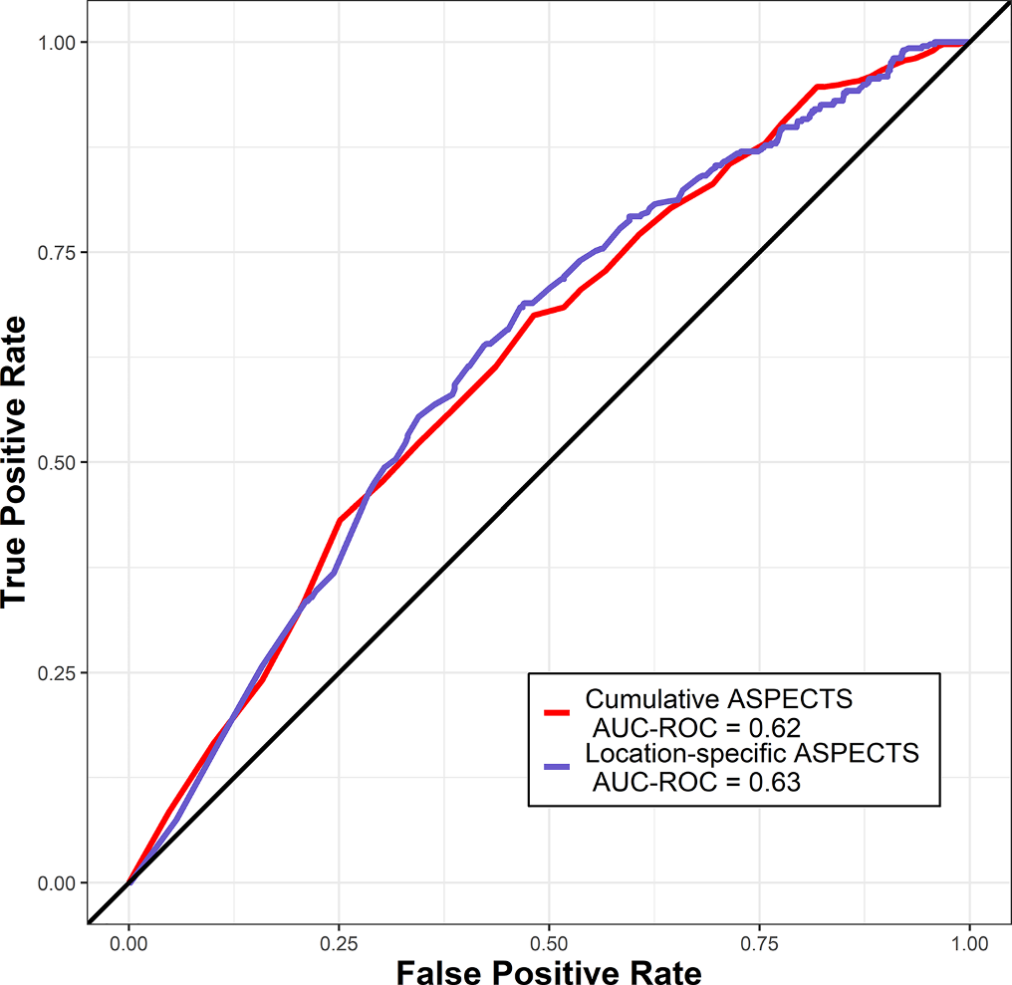
Area under the receiver operating characteristics curve (AUC-ROC) for predicting good clinical outcome (defined as modified Rankin scale of 0–2 after 90 days) after endovascular treatment using logistic regressions with either the cumulative ASPECTS (*red*) or the location-specific ASPECTS (*blue*)

Overall accuracy to predict good clinical outcome using a generalized linear model did not show a difference between i) cumulative ASPECTS (ACC = 0.619, 95% CI 0.58–0.64) and ii) location-specific ASPECTS (ACC = 0.629, 95% CI 0.60–0.65; *p* = 0.933). The results of the main machine-learning approach are summarized in Table 4 and Fig. 2.

**Table 4:** Comparison of predictive accuracies (95% CI) of machine-learning gradient boosting machine models predicting binarized clinical outcomes using either the cumulative ASPECTS or the location-specific ASPECTS for all patients (*n* = 1109). ROC-AUC values were compared using deLong’s test

Outcome definition (binarized)	Predictive accuracy (95% CI) using cumulative ASPECTS	Predictive accuracy (95% CI) using location-specific ASPECTS	Comparison of ROC-AUC (*p*-value)
mRS 0–1 after 90 days	0.62 (0.61–0.63)	0.62 (0.59–0.65)	0.470
mRS 0–2 after 90 days	0.63 (0.62–0.64)	0.61 (0.60–0.62)	0.496
mRS 0–3 after 90 days	0.64 (0.62–0.65)	0.63 (0.62–0.64)	0.534
mRS 4–6 after 90 days	0.64 (0.63–0.65)	0.60 (0.59–0.61)	0.336
mRS 6 after 90 days	0.69 (0.68–0.70)	0.69 (0.68–0.70)	0.988

*CI* confidence interval, *ASPECTS* Alberta Stroke Programme Early CT Score, *mRS* modified Rankin Scale, *ROC-AUC* receiver operating curve characteristics, *AUC* area under the curve

### Supplemental Results

The results of the additional analyses are presented in the online-only supplemental results. In brief, more extensive analyses, including the evaluation of various binarized and linear clinical outcomes (comprising the continuous spectrum of mRS and NIHSS and degree of recanalization as well as effects of laterality, see supplemental Tables S1–S3), as well as the application and comparison of machine-learning models (see supplemental Tables S4 and S5), showed no prognostic superiority using location-specific ASPECTS compared with cumulative ASPECTS.

## Discussion

Reliable assessment of infarct size and the associated neurological relevance is crucial for outcome prediction in both clinical studies and clinical practice. The ASPECTS has been used as established marker for evaluation of acute infarcts in multiple trials and daily practice. As ASPECTS does not weight individual brain regions according to their functional relevance or consider laterality, several previous studies attempted to improve the prognostic meaning by a location-specific approach [6].

Here, we assessed the original ASPECTS and several weighted variations for their prognostic performance in a large uniform stroke cohort. Our findings demonstrate that consideration of specific ASPECTS subregions does not improve overall prediction of clinical outcome. Although we were able to confirm differences in the impact of individual ASPECTS regions on the clinical outcome after EVT, seemingly these effects were not strong enough to translate into a more accurate prediction that would outperform the traditional cumulative ASPECTS.

Specifically, we found strongest effects for the insula followed by varying involvement of cortical areas (mostly M5 region) in our statistical approaches, including binary and linear outcomes for mRS after 90 days, as well as for NIHSS after 24 h using regression and machine-learning approaches. Our results are in line with the findings of previous studies that used advanced imaging techniques such as CT perfusion or diffusion-weighted imaging on MRI to assess the topographic correlation of infarct areas with poor clinical outcome after EVT [6]. The functional significance of the affected areas and their corresponding predictive importance has been highlighted in these studies. Compared to ASPECTS derived from non-contrast CT images, ASPECTS based on MR imaging and CT perfusion have even higher reliability and reproducibility, underscoring our findings [11]. In these analyses, the insula was the only region to be consistently linked to poor clinical outcome [5, 12–14], explained by its importance in recovery from aphasia or paralysis and by its association with autonomic functions [4, 15, 16]. Generally, infarct involvement of the insula indicates proximal occlusion of the MCA, which has been shown to be associated with worse clinical outcome and grade of recanalization than distal MCA occlusions with primarily cortical involvement [17].

However, our second most important ASPECTS area for poor clinical outcome was the peripheral M5 region. Likewise, this finding is reinforced by the results of previous studies that used advanced imaging modalities, which showed that the M5 region was the second most affected region after the insula [13, 14, 18]. Further studies have also been able to demonstrate the strong effect of the M5 region in non-contrast CT [19]. Additionally, in an analysis that investigated regional collateral status, it was reported that the collateral status of the M5 region was the only significant predictor of good functional outcome in comparison to all other cortical ASPECTS regions [20]. Here, good collateral supply appears to have a protective function, as the lateral cortical surface (M5) controls higher cortical functions such as language processing [21].

In addition to the individual contribution of the specific ASPECTS regions to the clinical outcome, the combinations of affected regions are of great importance; however, there are few data available in the existing literature about this topic. As expected from our initial results, the combination of both M5 and insula regions were found to be subset with the strongest two-way interactions terms with respect to the clinical outcome of all included patients. In a subgroup analysis of patients with poor baseline ASPECTS (< 6), strongest interactions were found for M1 and M6 regions as a two-way subset and M1 + M6 + insula regions as a three-way subset. It can be deduced that for proximal MCA occlusions, that were mainly included in this study, it is not necessarily the specific function of an individual area that is in the focus, but rather the regional extent and thus ultimately the infarct size, stretching from the central insula area to the peripheral M1 and M6 regions in unfavorable circumstances.

Machine-learning methods are becoming increasingly important in medical research and with good reason, as it has been shown that they can be superior to conventional statistical methods in the life sciences, specifically in outcome prediction after ischemic stroke [22]. We therefore decided to analyze our hypothesis not only with traditional logistic regressions, but mainly using a machine-learning approach. The hypothesis here was that the machine-learning models would receive the information from all the affected regions and could internally deduce which region would have a higher importance compared to the others in the overall setting. With respect to this approach on individual ASPECTS regions, the existing literature is also scarce, which makes it difficult to contextualize our findings. Nevertheless, the insula region also showed the highest impact on the clinical outcome by far, followed by the cortical regions in varying order depending on the clinical endpoint. Overall, the results of the machine-learning approach thus corroborate our findings in the logistic regressions, indicating that the overall extent of the early signs of ischemia, especially the combination of central and peripheral ASPECTS regions, is of highest importance. Still, the influence of the insula region on its own should not be overestimated, as the insula as the sole predictor performed significantly worse than the cumulative ASPECTS or the combination of all aspects regions, as indicated by our results.

There are some limitations to this study that we need to acknowledge. Although we analyzed a large dataset, our results only reflect the circumstances of a single institution and would benefit from external or multi-institutional validation. Furthermore, the validity of this study is limited by its retrospective design, although the stroke database was maintained in a prospective manner. Machine-learning models generally outperform conventional statistical methods in medical research for the purpose of outcome prediction. The use of a mixed-effect model could have been reasonable when using multiple variables that may influence each other. While there has been progress in incorporating mixed model effects into existing machine-learning approaches, these are not performed by default and were not part of the final analysis of this manuscript. As only patients with an EVT were included, the effects of selection bias must be considered. Moreover, endovascular treatment of included patients was mostly finished in shorter time windows below 6 h, and findings for patients in a longer onset to treatment time might show different results. Even though ASPECTS is a stable measure in the quantification of early signs of infarction, there is a certain variability in its evaluation. The evaluation by one radiologist in consensus with the e‑ASPECTS evaluation is therefore another limitation of this study.

In summary our results suggest that the functional differences in affected areas are not the main feature of native stroke imaging, but ultimately the extent of early signs of infarction. Consequently, the original ASPECTS assessment in LVO, defined as early as 2001, remains a stable and reliable predictor for clinical outcome and is not inferior to a weighted (location-specific) ASPECTS assessment. Nevertheless, we believe that future research with access to more and sharper data should continue to look for ways to enhance the original ASPECTS to provide an even better simple and reliable assessment of the early signs of infarction and thereby improve the treatment of acute stroke patients.

## Supplementary Information

Supplemental Methods, Supplemental Results and Supplemental Tables S1–S5
