# Supplementary material for: Location-specific ASPECTS does not improve Outcome Prediction in Large Vessel Occlusion compared to Cumulative ASPECTS
Source: Clin Neuroradiol. 2023 Jan 26;33(3):661–8. doi: 10.1007/s00062-022-01258-8 (PMC10449666; doi:10.1007/s00062-022-01258-8)
Supplement: Supplementary file 1 — Supplemental Methods, Supplemental Results and Supplemental Tables S1–S5 [file 62_2022_1258_MOESM1_ESM.pdf]

SUPPLEMENTAL MATERIAL

SUPPLEMENTAL METHODS

Comparison of cumulative and location-specific ASPECTS using machine-learning driven predictive models on clinical outcome

The performance to predict clinical outcome with machine-learning classifiers has been shown to be more accurate than conventional statistical models, such as logistic regressions. Therefore, machine-learning driven algorithms were used to assess the predictive performance of a location-specific ASPECTS, comprised of 10 binary parameters representing each ASPECTS region. The rationale here was that the different importance of the regions would be freely derived by the machine-learning model during optimization of hyper-parameters and then considered in the prediction. Gradient boosting algorithms were used as the main method. They are an extremely popular machine learning algorithm that have proven successful across many domains and are one of the leading methods for winning competitions on Kaggle (the most well-known online platform for predictive modeling and analytics). These algorithms produce a prediction model in the form of an ensemble of weak prediction models, typically decision trees. Gradient boosting thereby starts with a weak model (a decision tree with only a few splits) and sequentially boosts its performance by continuing to build new trees, where each new tree in the sequence tries to fix up where the previous one made the biggest mistakes (i.e., each new tree in the sequence will focus on the training rows where the previous tree had the largest prediction errors). We used the “caret” package in R (Kuhn, M. (2008). Building Predictive Models in R Using the caret Package. Journal of Statistical Software, 28(5), 1 - 26. doi:http://dx.doi.org/10.18637/jss.v028.i05) for generating the gradient boosting

classifiers. We also applied synthetic minority over-sampling (SMOTE), which downsamples the majority class and synthesizes new data points in the minority class to overcome potential negative impact on the model fit due to presence of class imbalances.

The dataset was partitioned into a training set (70%) and a test set (30%). Machine-learning models to predict clinical outcomes i) mRS 0 – 1 after 90 days, ii) mRS 0 – 2 after 90 days, iii) mRS 0 – 3 after 90 days, iv) mRS 4 – 6 after 90 days and v) mRS 6 after 90 days were performed for both the original, cumulative ASPECTS as well as the location-specific ASPECTS. The predictive performance of all models was assessed by obtaining a confusion matrix of the estimated and the actual values. Area under the curves of the respective receiver operating characteristics were compared for all outcomes between the cumulative and the location-specific ASPECTS using deLong's test.

Comparison of cumulative and location-specific ASPECTS using machine-learning driven predictive models on *categorical* clinical outcome in subgroups

Machine-learning models to predict clinical outcomes i) mRS 0 – 1 after 90 days, ii) mRS 0 – 2 after 90 days, iii) mRS 0 – 3 after 90 days, iv) mRS 4 – 6 after 90 days and v) mRS 6 after 90 days were performed for both the original, cumulative ASPECTS as well as the location-specific ASPECTS for the subgroups with either i) left-sided stroke, ii) right-sided stroke, and iii) patients with complete recanalization (indicated by mTICI 2c or 3) and without symptomatic hemorrhage in follow-up imaging. Due to smaller cohort sizes, these analyses were performed using cross-validation (using five folds), instead of separated training and testing. Area under the curves of the respective receiver operating characteristics were compared for all outcomes between the cumulative and the location-specific ASPECTS using deLong's test.

Results are outlined in Supplemental Tables S1-3.

Comparison of cumulative and location-specific ASPECTS using machine-learning driven predictive models on *linear* clinical outcome

Machine-learning driven linear modelling was performed for both cumulative and location-specific ASPECTS in linear outcomes for i) mRS after 90 days (0 – 6), ii) NIHSS after 24 hours (0 – 42), iii) shift between NIHSS at baseline and 24 hours after therapy and iv) shift between premorbid mRS and mRS after 90 days. Adjusted R-squared of machine-learning driven linear regressions were estimated and compared between cumulative ASPECTS and location-specific ASPECTS for each outcome, respectively, using ANOVA.

Results are outlined in Supplemental Table S4.

Comparison of machine learning algorithms to predict clinical outcome

To investigate the possibility that other machine-learning algorithms might perform better in predicting clinical outcome than the main method chosen (gradient boosting machines), it was compared with a selection of other commonly used machine-learning algorithms in the accuracy to predict good clinical outcome (mRS of 0 – 2 after 90 days). Further algorithms chosen comprised of eXtreme gradient boosting, random forest, k-nearest neighbor, glmnet regression and support vector machines.

Results are outlined in Supplemental Table S5.

Finding the subset of affected ASPECTS regions with strongest interactions

To find subsets ASPECTS regions with combined strongest effects on clinical outcomes, highest two-way and three-way interactions were assessed in a logistic regression model by applying a feasible solution algorithm, using the rFSA package in R. The feasible solution algorithm searches a data space for models of a user-specified form that are statistically optimal under a measure of quality (e. g. AIC).

Results are summarized in the Supplemental Results.

SUPPLEMENTAL RESULTS

Finding the subset of affected ASPECTS regions with strongest interactions

Subsets with strongest interaction between ASPECTS regions were found for i) insula * m5 (AIC = 722) for two-way interaction, ii) insula * m5 * lentiform nucleus (AIC = 719) for three-way interactions and iii) insula * M5 * lentiform nucleus * M3 (AIC = 715) for four-way interactions.

SUPPLEMENTAL TABLES

Supplemental Table S1 Comparison of predictive accuracies (95% CI) of machine-learning gradient boosting machine models predicting binarized clinical outcomes using either the cumulative ASPECTS or the location-specific ASPECTS in a subgroup of patients with right-sided stroke (n = 531). ROC-AUC values were compared using deLong's test.

**Outcome definition (binarized)**	**Predictive accuracy (95% CI) using cumulative ASPECTS**	**Predictive accuracy (95% CI) using location-specific ASPECTS**	**Comparison of ROC-AUC (p-value)**
mRS 0 – 1 after 90 days	0.61 (0.60 – 0.62)	0.60 (0.58 – 0.61)	0.941
mRS 0 – 2 after 90 days	0.61 (0.60 – 0.63)	0.61 (0.59 – 0.62)	0.877
mRS 0 – 3 after 90 days	0.63 (0.62 – 0.63)	0.62 (0.61 – 0.64)	0.542
mRS 4 – 6 after 90 days	0.64 (0.63 – 0.65)	0.63 (0.62 – 0.64)	0.309
mRS 6 after 90 days	0.70 (0.68 – 0.70)	0.69 (0.68 – 0.70)	1.000

CI = confidence interval; ASPECTS = Alberta Stroke Program Early CT Score; mRS = modified Rankin Scale; ROC-AUC = receiver operating curve characteristics; AUC = area under the curve

Supplemental Table S2 Comparison of predictive accuracies (95% CI) of machine-learning gradient boosting machine models predicting binarized clinical outcomes using either the cumulative ASPECTS or the location-specific ASPECTS in a subgroup of patients with left-sided stroke (n = 578). ROC-AUC values were compared using deLong's test.

**Outcome definition (binarized)**	**Predictive accuracy (95% CI) using cumulative ASPECTS**	**Predictive accuracy (95% CI) using location-specific ASPECTS**	**Comparison of ROC-AUC (p-value)**
mRS 0 – 1 after 90 days	0.65 (0.64 – 0.66)	0.64 (0.63 – 0.65)	0.746
mRS 0 – 2 after 90 days	0.66 (0.65 – 0.67)	0.64 (0.63 – 0.65)	0.507
mRS 0 – 3 after 90 days	0.64 (0.62 – 0.65)	0.63 (0.62 – 0.64)	0.643
mRS 4 – 6 after 90 days	0.64 (0.63 – 0.65)	0.61 (0.60 – 0.62)	0.243
mRS 6 after 90 days	0.69 (0.68 – 0.70)	0.70 (0.69 – 0.70)	1.000

CI = confidence interval; ASPECTS = Alberta Stroke Program Early CT Score; mRS = modified Rankin Scale; ROC-AUC = receiver operating curve characteristics; AUC = area under the curve

Supplemental Table S3 Comparison of predictive accuracies (95% CI) of machine-learning gradient boosting machine models predicting binarized clinical outcomes using either the cumulative ASPECTS or the location-specific ASPECTS in a subgroup of patients that attained complete recanalization (defined, as mTICI 2c or 3) and did not have a symptomatic hemorrhage in follow-up imaging (n = 508). ROC-AUC values were compared using deLong's test.

**Outcome definition (binarized)**	**Predictive accuracy (95% CI) using cumulative ASPECTS**	**Predictive accuracy (95% CI) using location-specific ASPECTS**	**Comparison of ROC-AUC (p-value)**
mRS 0 – 1 after 90 days	0.63 (0.61 – 0.64)	0.62 (0.61 – 0.64)	0.999
mRS 0 – 2 after 90 days	0.60 (0.59 – 0.62)	0.58 (0.57 – 0.60)	0.899
mRS 0 – 3 after 90 days	0.61 (0.60 – 0.62)	0.61 (0.60 – 0.62)	0.999
mRS 4 – 6 after 90 days	0.59 (0.58 – 0.61)	0.57 (0.55 – 0.58)	0.998
mRS 6 after 90 days	0.69 (0.67 – 0.70)	0.70 (0.69 – 0.73)	0.999

CI = confidence interval; ASPECTS = Alberta Stroke Program Early CT Score; mRS = modified Rankin Scale; ROC-AUC = receiver operating curve characteristics; AUC = area under the curve

Supplemental Table S4 Comparison of adjusted R-squared derived by machine-learning driven generalized linear models predicting the complete range of clinical outcomes using either the cumulative ASPECTS or the location-specific ASPECTS for all patients (n = 1109). Models were compared using ANOVA.

**Outcome definition (linear)**	**Adjusted R-squared using cumulative ASPECTS**	**Adjusted R-squared using location-specific ASPECTS**	**ANOVA (p-value)**
mRS after 90 days	0.0442	0.0443	0.443
NIHSS after 24 hours	0.0501	0.0522	0.232
Shift of baseline NIHSS to NIHSS after 24 hours	0.0167	0.0146	0.576
Shift of premorbid mRS to mRS after 90 days	0.074	0.070	0.880

CI = confidence interval; NIHSS = National institute of Health Stroke Scale; ASPECTS = Alberta Stroke Program Early CT Score; mRS = modified Rankin Scale

Supplemental Table S5 Comparison of machine learning algorithms to predict good clinical outcome (mRS of 0 – 2 after 90 days) using either the cumulative ASPECTS or the location-specific ASPECTS for all patients (n = 1109). Inferential assessment of model performance was used by analyzing if the pair-wise differences for each metric equals zero.

Machine Learning Algorithm	Predictive accuracy (95% CI) using cumulative ASPECTS	Predictive accuracy (95% CI) using composite ASPECTS	Inferential assessment of model performance
Gradient boosting machine	0.63 (0.62 – 0.64)	0.61 (0.60 – 0.62)	p = 0.876
XGBoost	0.63 (0.61 – 0.65)	0.63 (0.61 – 0.65)	p = 0.874
Random forest	0.61 (0.60 – 0.63)	0.62 (0.60 – 0.64)	p = 0.591
k-nearest neighbor	0.62 (0.60 – 0.64)	0.62 (0.60 – 0.64)	p = 0.133
glmnet	0.63 (0.61 – 0.65)	0.63 (0.61 – 0.65)	p = 0.811
Support vector machine	0.63 (0.61 – 0.65)	0.62 (0.60 – 0.64)	p = 0.905

NIHSS = National institute of Health Stroke Scale; ASPECTS = Alberta Stroke Program Early CT Score; mRS = modified Rankin Scale; XGBoost = Extreme Gradient Boost; glmnet = Lasso and Elastic-Net Regularized Generalized Linear Model
